# Cost-Effectiveness of Dabigatran versus Genotype-Guided Management of Warfarin Therapy for Stroke Prevention in Patients with Atrial Fibrillation

**DOI:** 10.1371/journal.pone.0039640

**Published:** 2012-06-22

**Authors:** Joyce H. S. You, Kia K. N. Tsui, Raymond S. M. Wong, Gergory Cheng

**Affiliations:** 1 Centre for Pharmacoeconomics Research, School of Pharmacy, Faculty of Medicine, The Chinese University of Hong Kong, Shatin, Hong Kong, China SAR; 2 Division of Hematology, Department of Medicine and Therapeutics, Faculty of Medicine, The Chinese University of Hong Kong, Shatin, Hong Kong, China SAR; Royal College of Surgeons, Ireland

## Abstract

**Background:**

Dabigatran is associated with lower rate of stroke comparing to warfarin when anticoagulation control is sub-optimal. Genotype-guided warfarin dosing and management may improve patient-time in target range (TTR) and therefore affect the cost-effectiveness of dabigatran compared with warfain. We examined the cost-effectiveness of dabigatran versus warfarin therapy with genotype-guided management in patients with atrial fibrillation (AF).

**Methodology/Principal Findings:**

A Markov model was designed to compare life-long economic and treatment outcomes of dabigatran (110 mg and 150 mg twice daily), warfarin usual anticoagulation care (usual AC) with mean TTR 64%, and genotype-guided anticoagulation care (genotype-guided AC) in a hypothetical cohort of AF patients aged 65 years old with CHADS_2_ score 2. Model inputs were derived from literature. The genotype-guided AC was assumed to achieve TTR = 78.9%, adopting the reported TTR achieved by warfarin service with good anticoagulation control in literature. Outcome measure was incremental cost per quality-adjusted life-year (QALY) gained (ICER) from perspective of healthcare payers. In base-case analysis, dabigatran 150 mg gained higher QALYs than genotype-guided AC (10.065QALYs versus 9.554QALYs) at higher cost (USD92,684 versus USD85,627) with ICER = USD13,810. Dabigatran 110 mg and usual AC gained less QALYs but cost more than dabigatran 150 mg and genotype-guided AC, respectively. ICER of dabigatran 150 mg versus genotype-guided AC would be >USD50,000 (and genotype-guided AC would be most cost-effective) when TTR in genotype-guided AC was >77% and utility value of warfarin was the same or higher than that of dabigatran.

**Conclusions/Significance:**

The likelihood of genotype-guided anticoagulation service to be accepted as cost-effective would increase if the quality of life on warfarin and dabigatran therapy are compatible and genotype-guided service achieves high TTR (>77%).

## Introduction

Warfarin was shown to effectively reduce risk of ischemic stroke in patients with atrial fibrillation (AF) [1]. The anticoagulation effect of warfarin, measured by the international normalized ratio (INR), is subject to wide inter- and intra-individual variability that possibly leads to hemorrhagic events despite careful dosage titration [2]. There is continuing search of new anticoagulants for safe and effective stroke prevention in patients with AF. Recently, results of the Randomized Evaluation of Long-Term Anticoagulation Therapy (RE-LY), a multi-centered trial including over 18,000 patients with AF, demonstrated that dabigatran (an oral direct thrombin inhibitor) 110 mg twice daily was associated with lower major bleeding and similar effectiveness in stroke prevention when compared with warfarin [3]. At higher dose (150 mg twice daily), dabigatran was associated with lower rate of stroke and similar major bleeding rate comparing to warfarin. Dabigatran at both doses were associated with higher risk of myocardial infarction and dyspepsia. An analysis of the RE-LY data showed a trend of better relative performance for dabigatran in centers with lower mean patient-time in therapeutic range (TTR), despite the differences in stroke and intracranial bleeding among centers in different TTR quartiles were not statistically significant [4]. Dabigatran 150 mg (twice daily) was more cost-effective than dabigatran 110 mg or warfarin therapy [5] unless the TTR with warfarin was >72.6% [6].

Warfarin therapy with good INR control (TTR>75%) is associated with lower event rates, yet the majority of patients on warfarin achieve only suboptimal INR control [1]. Association of warfarin pharmacogenetics (*CYP2C9* and *VKORC1*genotypes) and dosage requirement was widely examined. Nevertheless, genotype-guided warfarin dosing algorithm alone did not achieve significant improvement in TTR at centers with high level of anticoagulation control [7]. Genotype-guided warfarin dosing was potentially cost-effective in practice sites with poor INR control or patients with high risk of bleeding [8]–[9]. Further clinical research demonstrated that providing patients’ genotype information to clinicians who managed the warfarin therapy was associated with reduction in hospitalization for major bleeding and thromboembolism [10]. Applying genotype data in both warfarin dosing and patient care therefore might optimize INR control.

Strategies to achieve good INR control (TTR>75%) with warfarin and using a direct thrombin inhibitor (dabigatran) have different economic and clinical implications for patients, clinicians and decision-makers to consider. The objectives of the present study were to evaluate the potential clinical and economic outcomes of genotype-guided management of warfarin and use of dabigatran in patients newly diagnosed with AF from the perspective of healthcare payers.

## Methods

### Decision Model

**Figure 1:**
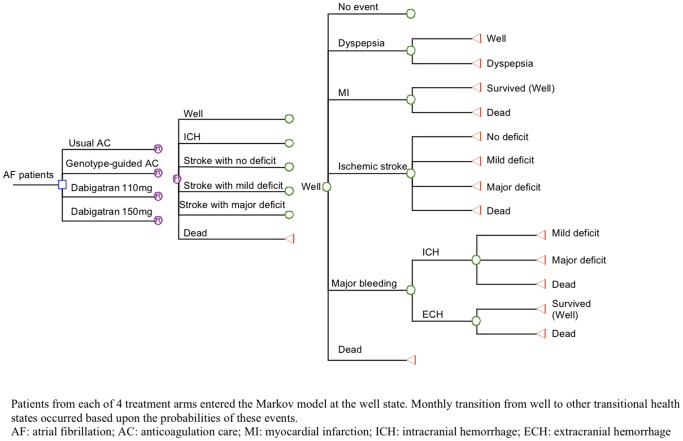
Markov model.

A Markov model (**Figure 1**) was designed to simulate the life-long outcomes of four anticoagulation treatment strategies in a hypothetical cohort of 65-year-old patients with newly diagnosed AF: (1) Standard warfarin dosing with usual anticoagulation care (usual AC), (2) genotype-guided warfarin dosing and management (genotype-guided AC), (3) initiation of dabigatran 110 mg twice daily, and (4) initiation of dabigatran 150 mg twice daily. Markov modeling is a form of decision analysis in which hypothetical patients proceed through health states over time based on probability inputs of the model. Patients of all treatment arms entered the model at the Markov health state of being well and transited to other health states (remained well, dyspepsia, myocardial infarction, ischemic stroke with major deficit, minor deficit or no residual deficit, intracranial hemorrhage, extra-cranial hemorrhage and dead) in the next cycle. Two tiers of outcomes were simulated for each study arm: Total direct medical cost and quality-adjusted life-years (QALYs) gained were calculated over a maximum period of 25 years with monthly cycle.

The patient selection criteria were adopted from those of the RE-LY trial [3]. Patients aged 65 years or above with a high risk for stroke (CHADS_2_ score of 2 or higher) were included. Exclusion criteria included presence of severe heart-valve disorders or severe stroke within 6 months. The warfarin dose in usual AC group would be adjusted to an INR of 2–3, and the INR would be monitored at least monthly thereafter. In the genotype-guided AC group, *CYP2C9* and *VKORC1* genotypes would be examined by in-house genotyping assay. The starting dose of warfarin would be designed by a dosing algorithm, using demographic, clinical and genetic (*CYP2C9* and *VKORC1* genotypes) data to target at INR 2–3 [11]. Patients with wild-type *CYP2C9* and *VKORC1* (normal warfarin sensitivity) would be followed up by usual anticoagulation care (at least monthly INR monitoring), whereas patients with genotypes of high or low warfarin sensitivity would be managed by intensified anticoagulation care (INR monitoring at least twice per month and patient education on warfarin therapy including impact of patient’s genotype on warfarin sensitivity and INR control). The INR control might be in, below or above the target range and patients might consequently experience bleeding or ischemic events. In dabigatran 110 mg and 150 mg groups, patients would be initiated with dabigatran 110 mg twice daily and 150 mg twice daily, respectively. Patients who survived ischemic stroke would change the initial anticoagulation therapy to dabigatran 150 mg twice daily. Those who survived major bleeding event would stop the current anticoagulation therapy and start on aspirin alone.

### Clinical Inputs

The clinical inputs of the model were retrieved from literature. Literature search on MEDLINE over the period 1990–2012 was performed using keywords “atrial fibrillation”, “warfarin”, “dabigatran”, “bleeding”, “thromboembolism”, “QALY”, “INR”, “genotyping”, “*VKORC1*” and “*CYP2C9*”. The selection criteria of clinical trials on warfarin and dabigatran treatment and related events were: (1) reports in English; (2) patients involved in the trials were at least 18 years of age; and (3) control of INR and/or the incidence of major events (bleeding or ischemic event) were reported. All articles retrieved by this process were screened for relevance to our model. Case reports were excluded. The preferred type of studies was meta-analysis. If multiple randomized controlled trials were available for the same model input, the pooled average would be derived from the studies weighted against the number of patients in each study and used as the base-case model input. If a model input was reported in both randomized and non-randomized trials, the pooled average from randomized trials would be used as base-case value whereas the range for sensitivity analysis would be derived from both randomized and non-randomized trials. If a model input was not reported in meta-analyses nor randomized controlled trials, it would be estimated from the findings of non-randomized controlled trials.

Clinical inputs were shown in **Table 1**. The mean TTR (64%) in warfarin group of the RE-LY trial [3] was used as the base-case TTR of patients in usual AC. Out-of-range INR was defined as <1.8 or >3.2. The prevalence of patients with normal warfarin sensitivity was retrieved from a prospective study [10]. In the RE-LY trial, the anticoagulation control at the study centers were stratified in four quartiles (TTR <57.1%, 57.1%–65.5%, 65.5%–72.6% and >72.6%) [4]. In those centers achieving good anticoagulation control (TTR>75%), the median TTR was 78.9% with inter-quartile range of 70.9%–86.7%. The genotype-guided AC was assumed to achieve good anticoagulation control with base-case TTR = 78.9%. The range for sensitivity analysis was extended to 65%–100% in order to examine the level of TTR required for genotype-guided AC to be cost-effective.

**Table 1:** Model inputs.

Variables	Base-case value			Range	References
***INR control on Warfarin***					
Percentage of in-range time in usual AC	64%			57%–65.5%	3–4
Percentage of in-range time in genotype-guided AC	78.9%			65%–100%	4
Proportion of below-range time among out-of-range time	52%			42%–62%	1
Prevalence of patients with normal warfarin sensitivity	29.2%			23.4%–35.0%	10
***Stroke***					
Rate of ischemic stroke: warfarin at in-range INR (per patient year)	1.3%			0.5%–1.6%	12
Relative risk of ischemic stroke: warfarin at below-range INR	1.70			1.70–6.88	12
Relative risk of ischemic stroke: warfarin at above-range INR	1				Assumption
Rate of ischemic stroke: aspirin (per patient year)	2.7%			0.8%–13.7%	14
Relative risk of stroke: dabigatran 150mg vs warfarin	0.76			0.59–0.97	3, 34
Relative risk of stroke: dabigatran 110mg vs warfarin	1.11			0.88–1.39	3, 34
Ischemic stroke on warfarin or dabigatran (%)					3,13,15
Fatal (within 30 days)	8.2%			8.2%–10.1%	
Major deficit	40.2%			40.2%–41.7%	
Minor deficit	42.5%			34.8%–42.5%	
No residual deficit	9.1%			9.0%–13.3%	
Ischemic stroke on aspirin (%)					3,13,15
Fatal (within 30 days)	17.9%			10.1–17.9	
Major deficit	30%			30.0–41.7	
Minor deficit	41%			34.8–41.0	
No residual deficit	11%			11.0–13.3	
***Major Bleeding***					
Rate of major bleeding: warfarin at in-range INR (per patient year)	1.5%			1.0%–1.5%	12
Relative risk of major bleeding: warfarin at above-range INR	8.28			3.21–8.28	12
Relative risk of major bleeding occurred at below-range INR	1			–	Assumption
Relative risk of major bleeding: aspirin vs warfarin	0.64			0.5–0.8	16–17
Relative risk of major bleeding: dabigatran 150mg vs warfarin	0.93			0.81–1.07	3, 34
Relative risk of major bleeding: dabigatran 110mg vs warfarin	0.80			0.70–0.93	3, 34
Proportion of ICH in major bleeding					
Warfarin	22%			18%–25%	3–4
Dabigatran 150mg	12.6%			6.3%–13.4%	4
Dabigatran 110mg	8.9%			4.6%–11.9%	4
Aspirin	21%			16%–25%	16–17
Mortality rate of ICH	48.6%			36%–61%	18–19
Mortality rate of ECH	5.1%			0.1%–10.1%	18
***MI***					
Rate of MI (per patient year)					
Warfarin	0.64%			0.51%–0.77%	3,34
Aspirin	0.53%			0.40%–0.60%	39–40
Dabigatran 150mg	0.81%			0.65%–0.97%	3,34–35
Dabigatran 110mg	0.82%			0.66%–0.98%	3,34–35
Mortality rate of MI	15%			10.3–24.6%	41
***Utility inputs***					
Warfarin therapy	0.95	0.95–1			20–22
Dabigatran therapy	1.00	0.95–1			Assumption,42
Aspirin					
Major bleeding					
Intracranial	0.51	0.15–0.85			20–22
Extracranial	0.80	0.79–0.84			21–22
Ischemic stroke					
Major deficit	0.39	0–0.50			20–22
Minor deficit	0.75	0.50–0.99			20–22
Myocardial infarction	0.84	0.67–0.96			23
Dyspepsia	0.97	0.74–0.98			24
***Cost inputs (USD)***					
Genotyping	72	50–200			32
Monthly cost of usual AC per patient	31	21–36			29–30
Increment factor of monthly cost of intensified AC	2	2–3			Assumption
Monthly cost of warfarin	6	4–20			31
Monthly cost of dabigatran 110mg twice daily	240	200–270			Assumption
Monthly cost of dabigatran 150mg twice daily	240	200–270			31
One-time cost of major event					25
ICH	45,959	21,675–55,151			
ECH	23,798	17,445–39,308			
Ischemic stroke					
Moderate to severe	65,984	53,243–78,724			
Mild	44,043	35,234–52,852			
TIA	19,514	15,611–23,417			
Myocardial infarction					
Survived	27,996	20,945–43,727			
Dead	20,654	14,447–44,498			
Monthly cost					25–28
ICH	5,740	2,100–10,000			
Ischemic stroke with major deficit	5,430	2,100–9,000			
Ischemic stroke with mild deficit	2,500	1,000–4,300			
ICH and ischemic stroke	7,280	3,180–13,790			

The rates of major bleeding (including intracranial and extracranial hemorrhage) and ischemic stroke in therapeutic range of INR (INR ≤ 3 and INR ≥ 2) and the risks for stroke in under-coagulated patients (INR <2) and major bleeding in over-coagulated patients (INR>3) were estimated in a meta-analysis of outcomes of warfarin anticoagulation for atrial fibrillation [12]. The risk of major bleeding in under-coagulated patients and major thromboembolic events in over-coagulated patients were both assumed to be the same as patients with in-range INRs.

The relative risks of major bleeding and ischemic event and rates of myocardial infarction of dabigatran groups, comparing to usual AC were derived from the results of RE-LY [3]. The rates of major bleeding and ischemic events in dabigatran arms were estimated from the relative risk of event of dabigatran versus usual AC, and the rate of major events in usual AC. The rate of ischemic stroke and percentage of ischemic strokes with major, minor or no deficit on aspirin were derived from prospective trials [3], [13]–[15]. The rate of major bleeding on aspirin was estimated from relative risk of bleeding on aspirin versus warfarin and the bleeding rate in usual AC [16]–[17]. The mortality rates of intracranial hemorrhage, extracranial hemorrhage, ischemic stroke and acute myocardial infarction within 30 days of event were estimated from observational studies [15], [18]–[19].

### Utility and Cost Inputs

The QALYs gained in each study arm were estimated from the utility scores of different health states (remained well on warfarin, dabigatran or aspirin, dyspepsia, myocardial infarction, major neurologic deficit, mild neurologic deficit, no neurologic deficit, extra-cranial hemorrhage and dead) and the time spent in each state (**Table 1**) [20]–[24]. The QALYs were discounted with a rate of 3% annually. The one-time treatment cost and monthly cost of major events (extracranial hemorrhage, intracranial hemorrhage, stroke and myocardial infarction) were estimated from the perspective of healthcare payers [25]–[28]. The monthly cost of usual anticoagulation care management, including staff time, laboratory tests and administrative cost, was estimated from economic analyses on anticoagulation care [29]–[30]. The cost of the hypothetical intensified anticoagulation service was assumed to be 2-fold (ranging 2- to 3-fold) of the usual anticoagulation service cost. The monthly warfarin drug cost was estimated from retail pricing of generic warfarin [31]. The cost of *CYP2C9* and *VKORC1* genotyping assay was estimated from literature [32]. The cost of dabigatran was retrieved from retail pricing [31]. All costs were discounted to 2012 costs with an annual rate of 3%.

### Model Validation, Cost-effectiveness Analysis and Sensitivity Analysis

In the present model, the rates of major bleeding and ischemic stroke in the warfarin therapy arms were estimated from the average individual TTR and the risk of event in patients with out-of-range INR. The event rates in dabigatran groups were simulated by relative risks of events in dabigatran versus warfarin. The event rate of genotype-guided AC was estimated using assumed improvement in TTR. The predictive validity of the model in the above study arms was assessed by comparing the model results with the clinical trial results.

A treatment strategy was dominated when it was more costly and gained less QALYs than another treatment option. The incremental cost per QALY gained (ICER) of each arm (excluding the dominated strategy), comparing to the next less costly arm, was calculated using the following equation: Δcost/ΔQALYs. Using the threshold of USD50,000 as the willingness-to-pay per QALY, the most effective strategy with ICER USD50,000 or less was considered as cost-effective [33].

Sensitivity analysis was performed by TreeAge Pro 2009 (TreeAge Software, Inc., Williamstown, MA, USA) and Microsoft Excel 2007 (Microsoft Corporation, Redmond, WA, USA) to examine the robustness of the model results to variation of all parameters. Threshold values of influential factors were identified by one-way sensitivity analysis over the high/low values. To evaluate the impact of the uncertainty in all variables simultaneously, a probabilistic sensitivity analysis was performed using Monte Carlo simulation. The cost and QALYs of each study arm were recalculated 10,000 times by simultaneously varying the values of each model input through the ranges of sensitivity analysis.

## Results

### Model Validation

The predictive validity of model was examined by comparing the rates of major bleeding and ischemic stroke (per 100 patient years) simulated by the model with the actual reported event rates in RE-LY trial [3], [34]. In the base-case scenario, patients in usual AC with mean TTR of 64% had major bleeding (3.39%) and ischemic stroke (1.35%) rates, similar to those in RE-LY trial (3.57% and 1.20% respectively). Comparing to the RE-LY findings, the simulated bleeding rates of dabigatran 110 mg (2.71% vs 2.87%) and dabigatran 150 mg (3.15% vs 3.32%) as well as simulated ischemic stroke rates of dabigatran 110 mg (1.51% vs 1.34%) and dabigatran 150 mg (1.03% vs 0.92%) were compatible to the reported event rates. The genotype-guided AC was hypothesized to achieve TTR 78.9% in the base-case scenario and the total simulated event rate of genotype-guided AC (bleeding 2.21% and ischemic stroke 1.27%) reduced by 26% when compared to usual AC. It was similar to the reported reduction in hospitalization rate (by 28%) for bleeding or thromboembolism associated with providing patients’ warfarin genotyping data with interpretations to clinicians in a prospective study [10].

### Cost-Effectiveness Analysis

The base-case analysis (**Table 2**) showed that both dabigatran arms gained higher QALYs than warfarin arms with higher cost. Dabigatran 110 mg gained less QALYs but cost more than dabigatran 150 mg and therefore dabigatran 110 mg was dominated by dabigatran 150 mg. Similarly, genotype-guided AC dominated usual AC. After excluding the two dominated options, the ICER of dabigatran 150 mg was USD13,810 when compared with genotype-guided AC. Using the threshold of USD50,000 as the willingness-to-pay per QALY, dabigatran 150 mg was the most cost-effective option in the base-case scenario.

**Table 2:** Expected Cost and QALYs in Base-case Analysis.

Strategy	Cost (USD)	QALYs	ICER^a^ (USD) vs genotype-guided AC
Genotype-guided AC^b^	85,627	9.554	–
Usual AC	90,481	9.444	Dominated by genotype-guided AC
Dabigatran 150mg	92,684	10.065	13,810
Dabigatran 110mg	102,536	10.026	Dominated by dabigatran 150mg

a: The incremental cost per QALY gained (ICER) of each arm (excluding the dominated strategy), comparing to the next less costly arm, was calculated using the following equation: Δcost/ΔQALYs. Using the threshold of USD50,000 as the willingness-to-pay per QALY, the most effective strategy with ICER USD50,000 or less was considered as cost-effective.

b: AC = Anticoagulation care.

**Figure 2:**
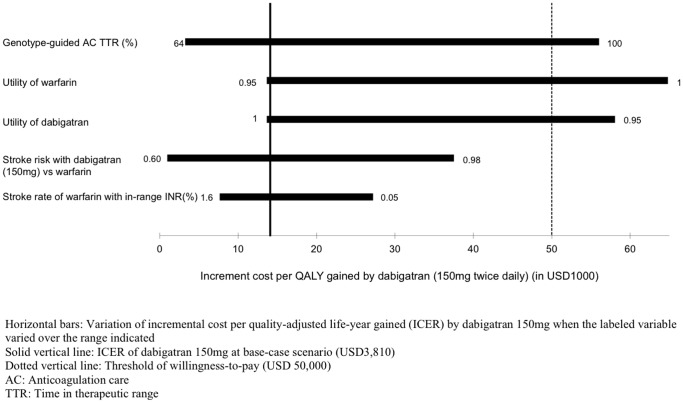
One-way sensitivity analysis on incremental cost per quality-adjusted life-year (ICER) gained by dabigatran 150mg.

Five model inputs were shown to be influential to the ICER of dabigratan 150 mg in one-way sensitivity analysis: (1) average TTR in genotype-guided AC, (2) utility of warfarin, (3) utility of dabigatran, (4) risk of stroke with dabigatran 150 mg versus warfarin and (5) stroke rate with warfarin when INR was in target range. **Figure 2** showed the variation of ICER of dabigatran 150 mg over the ranges of these five variables. The ICER of dabigatran 150 mg would become >USD50,000 (and genotype-guided AC would become the most cost-effective option) when average TTR in genotype-guided AC was extended over 98%, utility of warfarin therapy was ≥0.99, or utility of dabigatran therapy was ≤0.95. The change of stroke rate with warfarin or risk of stroke with dabigatran would vary the ICER of dabigatran 150 mg from less than USD10,000 to as high as USD38,000.

**Figure 3:**
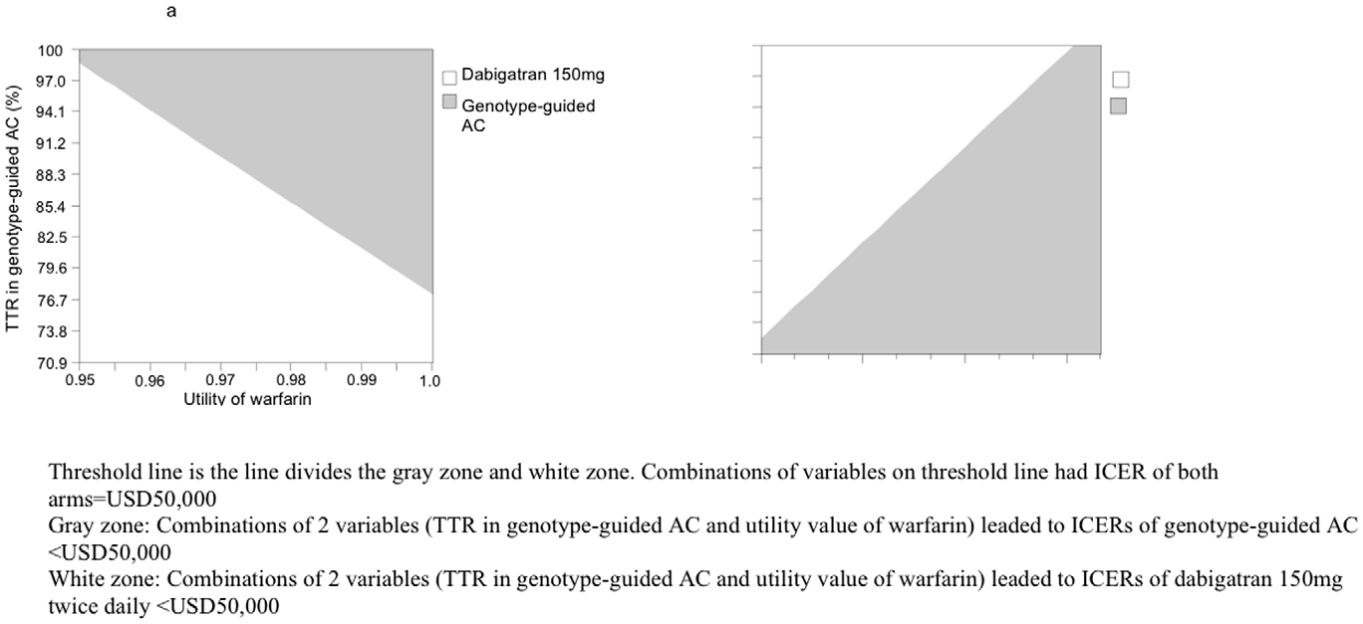
Two-way sensitivity analyses on the cost-effectiveness of dabigatran 150mg versus genotype-guided anticoagulation care (AC).

Two-way sensitive analyses (**Figures 3a, b**) were conducted to further examine the impact of variation of warfarin utility versus TTR in genotype-guided AC, and warfarin utility versus dabigatran utility on the model results. The results (**Figure 3a** **)** indicated that, with utility of warfarin ranging between 0.95 to 1.0, genotype-guided AC would be the most cost-effective option if it achieved high TTR (77% to 98%). **Figure 3b** showed that the genotype-guided AC would be the most cost-effective option if utility value of warfarin is the same or higher than that of dabigatran.

**Figure 4:**
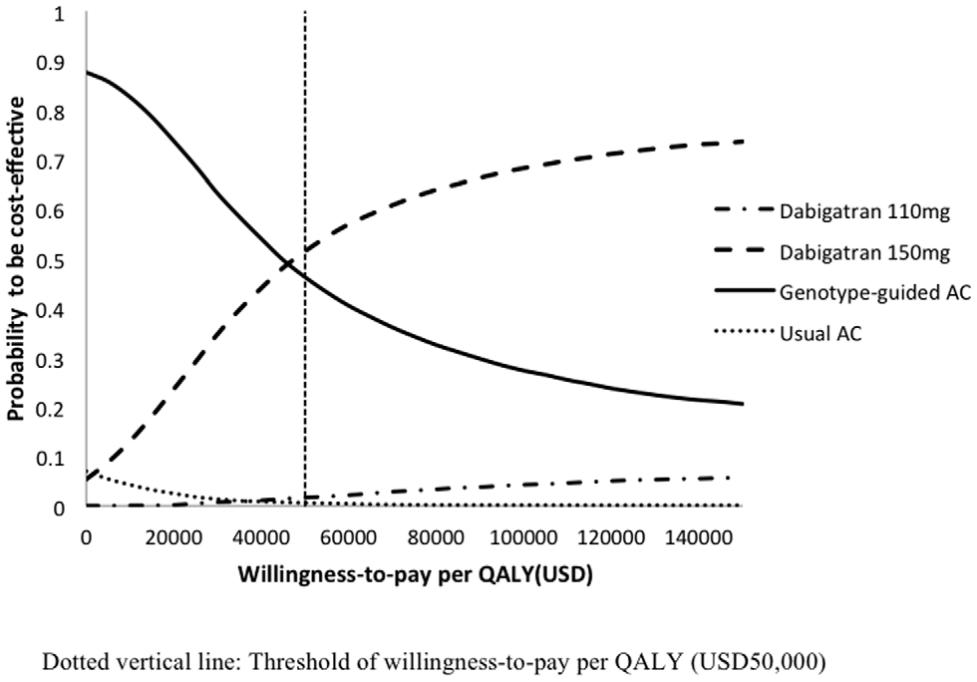
Variation in probability of each treatment option to be cost-effective against willingness-to-pay per QALY.

The probabilistic sensitivity analysis was performed by 10,000 Monte Carlo simulations. The probabilities of each strategy to be cost-effective were examined in the acceptability curve over a wide range of willingness-to-pay per QALY, from USD0-150,000 (**Figure 4**). Using USD50,000 as the threshold willingness-to-pay, dabigratan 150 mg was the most likely option to be cost-effective in 51.6% of time, whereas genotype-guided AC was cost-effective in 46.2% of time. The probabilities of usual AC and dabigatran 110 mg to be cost-effective were 0.6% and 1.6% of time, respectively. Dabigatran 150 mg was more costly (p<0.001) than genotype-guided AC with mean cost difference of USD10,416 (95%CI = 10,282–10,550) and gained higher QALYs (p<0.001) by mean QALYs difference = 0.217 (95%CI = 0.214–0.220).

## Discussion

Dabigatran 150 mg twice daily regimen was associated with lower rates of stroke for patients with AF, but similar rate of major bleeding comparing with warfarin therapy controlled at average TTR of 64% in the RE-LY trial. Recent meta-analysis has demonstrated that dabigatran is associated with increased risk of myocardial infarction [35]. Dabigatran may offer patients an option with less stringent monitoring requirements than warfarin therapy. Nevertheless, the high drug cost of dabigatran (approximately USD240 per month) would make it less affordable as life-long treatment for many patients. The choice of an anticoagulation service striving to achieve high TTR with warfarin therapy, or using oral direct thrombin inhibitor require qualifying and quantifying the impact of stroke prevention, risk of bleeding and acute coronary events.

In the present study, we assessed the potential life-long cost and effectiveness of applying pharmacogenetic data to guide the dosing and management of warfarin versus using two dabigatran regimens for newly diagnosed AF patients. In base-case analysis, dabigatran 150 mg was the preferred option with ICER (USD13,810) less than the threshold of willingness-to-pay per QALY (USD50,000). Our base-case results were consistent with the cost-effectiveness analyses comparing dabigatran with warfarin in the US and Canada settings [5], [36].

In the one-way sensitivity analysis, the variation of three variables increased the ICER of dabigatran 150 mg over USD50,000: TTR in genotype-guided AC and utility values of warfarin and dabigatran. Our findings showed that a slight variation of the utility values of the two drugs between 0.95 to 1.0 could change the choice of the most cost-effective option from dabigatran 150 mg to genotype-guided AC. It is believed that dabigatran, requiring less periodic blood testing and follow-ups, would have better quality of life (thus higher utility value) than warfarin therapy. The base-case utility value selected for dabigatran (1.0) was therefore higher than that of warfarin (0.95). Freeman et al reported the results of a cost-effectiveness analysis of dabigatran versus usual warfarin care that dabigatran was more cost-effective (ICER = USD45,372) when the base-case utility score of dabigatran was higher than that of warfarin (0.994 versus 0.987) [5]. These findings were consistent with results of our base-case analysis. Freeman et al also found the cost-effectiveness of dabigatran sensitive to the variation of utility values of the anticoagulants over a narrow range (0.95 to 1.0) in a similar manner to our two-way sensitivity analysis.

The 10,000 Monte Carlo simulations showed that the difference in QALYs between dabigatran 150 mg and genotype-guided AC was very narrow (0.217 QALYs  = 2.6 quality-adjusted months), implying that the life-long impact of stroke prevention and adverse events of both arms were compatible. The probabilities of dabigatran 150 mg and genotype-guided AC to be cost-effective were very similar (approximately 50%), possibly due to the impact on ICER as a result of small variation in utility values of the two drugs. In order to be better informed on the impact of patient quality of life while receiving different anticoagulants, health-related quality of life research comparing dabigatran versus warfarin is highly warranted.

We also explored the potential improvement in INR control required in order for genotype-guided AC to be cost-effective. The two-way sensitivity analysis showed that at high utility value of warfarin therapy (>0.98), genotype-guided AC would need to improve TTR from 64% in usual AC to >77% to become the most cost-effective option. Our results were similar to the cost-effectiveness findings reported by Shah and Gage that warfarin therapy would be more cost-effective than dabigatran when the average TTR was over 72.6% and the utility values of warfarin was high (0.987) [6].

The clinical benefits of warfarin pharmacogenetics remain uncertain. Most genotype-guided dosing algorithms only explain up to 46%–68% of dosage variation [11], [37]. It is also anticipated that impact of genotype-based dosage initiation on INR control would not be a long-term effect. Any long-term benefit of applying pharmacogenetics data should be generated from genotype-based triage of patients to intensified anticoagulation care. Epstein et al. reported findings of a prospective, comparative study that providing *CYP2C9* and *VKORC1* genotyping results with interpretation to physicians who managed warfarin therapy would reduce the hospitalization rate for bleeding or thrombembolism by 28%, when comparing to historical records [10]. The reduction in hospitalization was believed to be a combined effect of warfarin dosage adjustment and more vigilant care for warfarin-sensitive patients, yet the results was limited by the study design. Randomized controlled trials are required to investigate the benefits of applying pharmacogenetics to guide both dosing and care model of warfarin therapy. Possible interventions to apply genotype data to improve anticoagulation control include incorporating the knowledge of patients’ genotype in dosing, monitoring and patient education. Patients with low-dose genotype could be triaged to an intensive anticoagulation service with more frequent monitoring and in-depth patient education to emphasize the effect of genetic make-up on warfarin therapy [38].

This study is an example of decision analysis to compare the potential changes in economic and clinical outcomes of a dabigatran versus warfarin therapy with interventions to upgrade INR control. The results demonstrated a few influential factors (utility values of the two drugs and INR control required by genotype-guided AC to be cost-effective) which indicated the target values for dabigatran 150 mg or genotype-guided AC to be cost-effective, and therefore assisted clinicians to be better informed on the choice of anticoagulation therapy.

The present model was limited by projecting life-long events using key model inputs of dabigatran from a clinical trial of 2 years. Projecting life-long outcomes using short-term clinical trial data may weaken the robustness of the model findings. The cost items were limited to the resources of anticoagulation therapy and related complications. All the model inputs were examined in sensitivity analysis and probabilistic sensitivity analysis over a wide range to test for robustness of the results.

In conclusion, dabigatran 150 mg seems to be cost-effective (ICER <USD50,000) at centers with TTR ≤64%. The better INR control (measured by TTR) achieved by an anticoagulation center, the less cost-effective of dabigatran would become. The likelihood of genotype-guided anticoagulation service to be accepted as cost-effective would increase if the quality of life on warfarin and dabigatran therapy are compatible and genotype-guided service achieves high TTR (>77%). Further work is needed to better compare clinical outcomes and quality of life on dabigatran and genotype-guided warfarin management.
